# Strategies from UW-Madison for rescuing biomedical research in the US

**DOI:** 10.7554/eLife.09305

**Published:** 2015-06-30

**Authors:** Judith Kimble, William M Bement, Qiang Chang, Benjamin L Cox, Norman R Drinkwater, Richard L Gourse, Aaron A Hoskins, Anna Huttenlocher, Pamela K Kreeger, Paul F Lambert, Marsha R Mailick, Shigeki Miyamoto, Richard L Moss, Kate M O'Connor-Giles, Avtar Roopra, Krishanu Saha, Hannah S Seidel

**Affiliations:** Department of Biochemistry and Howard Hughes Medical Institute, University of Wisconsin–Madison, Madison, United States; Department of Zoology and Laboratory of Cellular and Molecular Biology, University of Wisconsin–Madison, Madison, United States; Laboratory of Genetics, Waisman Center and Department of Neurology, University of Wisconsin–Madison, Madison, United States; Department of Medical Physics, Morgridge Institute for Research and Laboratory for Optical and Computational Instrumentation, University of Wisconsin–Madison, Madison, United States; McArdle Laboratory for Cancer Research, University of Wisconsin–Madison, Madison, United States; Department of Bacteriology, University of Wisconsin–Madison, Madison, United States; Department of Biochemistry, University of Wisconsin–Madison, Madison, United States; Department of Medical Microbiology and Immunology and Department of Pediatrics, University of Wisconsin–Madison, Madison, United States; Department of Biomedical Engineering, University of Wisconsin–Madison, Madison, United States; McArdle Laboratory for Cancer Research, University of Wisconsin–Madison, Madison, United States; Waisman Center, University of Wisconsin–Madison, Madison, United States; McArdle Laboratory for Cancer Research and Cell Signaling Program, Carbone Cancer Center, University of Wisconsin–Madison, Madison, United States; School of Medicine and Public Health and Department of Cell and Regenerative Biology, University of Wisconsin–Madison, Madison, United States; Laboratory of Cellular and Molecular Biology, Laboratory of Genetics, and Laboratory for Optical and Computational Instrumentation, University of Wisconsin–Madison, Madison, United States; Department of Neuroscience, University of Wisconsin–Madison, Madison, United States; Department of Biomedical Engineering and Wisconsin Institute for Discovery, University of Wisconsin–Madison, Madison, United States; Department of Biochemistry, University of Wisconsin–Madison, Madison, United States

**Keywords:** NIH, science policy, careers in science, grad school, postdoc, funding

## Abstract

A cross-campus, cross-career stage and cross-disciplinary series of discussions at a large public university has produced a series of recommendations for addressing the problems confronting the biomedical research community in the US.

**DOI:** http://dx.doi.org/10.7554/eLife.09305.001

Systemic flaws threaten the future of biomedical research in the United States because the number of researchers competing for federal research funds is continuing to grow as the pool of real dollars available to support them continues to shrink. This imbalance has created a hypercompetitive research environment that endangers the vitality of biomedical science in the US. Last year a group of four prominent scientists—Bruce Alberts, Marc Kirschner, Shirley Tilghman and Harold Varmus—published a set of recommendations aimed at rectifying this imbalance (Alberts et al., 2014). These recommendations were subsequently vetted by a group of about 30 scientific leaders: these leaders agreed about the problems, but not the solutions (Alberts et al., 2015). One recommendation—cutting the number of PhD students in order to ultimately decrease the number of independent researchers—was especially controversial (Kelly and Marians, 2014; Marder, 2014).

To broaden the debate on these issues, the University of Wisconsin–Madison (UW-Madison) recently held a workshop, ‘Rescuing the US Biomedical Research Enterprise: Strategies and Pathways Ahead’. The goal of this workshop was to consider a range of ideas and generate additional recommendations to address the same basic dilemma. In contrast to previous efforts (McDowell et al., 2014; Alberts et al., 2015), this workshop solicited input from all sectors of the research community. Both the leaders of the workshop and the participants came from across campus, including the School of Medicine and Public Health, the College of Letters and Sciences, the College of Agriculture and Life Sciences, and the College of Engineering. Involvement also spanned career stages, including graduate students, postdocs, staff scientists, faculty of all ranks, and university administrators.

UW-Madison was an appropriate place for this broader discussion for a number of reasons: it is a large, publicly funded university with a long history of major contributions to biomedical research (such as vitamin D, reverse transcriptase and human embryonic stem cells); it is a pioneer in technology transfer, starting in 1925 with the founding of the Wisconsin Alumni Research Foundation; and it is a formidable center for graduate education (awarding about 200 PhDs per year in the biological sciences, defined broadly).

## How the UW-Madison workshop worked

**Figure 1.**
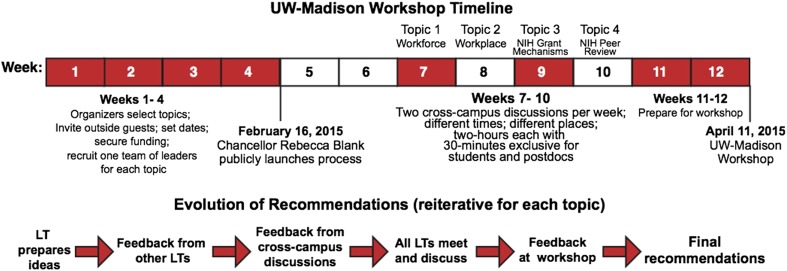
Process used for the UW-Madison workshop. The process started with the recruitment of four leadership teams (LTs), with four members for each team. The leadership teams led the cross-campus discussions during weeks 7–10 and presented the recommendations that emerged from these discussions at the April 11 workshop. **DOI:** http://dx.doi.org/10.7554/eLife.09305.002

Our approach was more of a workshop process than a typical workshop (Figure 1). A public launch was led by the Chancellor of UW-Madison, Rebecca Blank, and two of the present authors (MRM and JK). Campus-wide pre-workshop discussions were then held over the course of a month, each week focusing on a different topic: the funding mechanisms used by the National Institutes of Health (NIH); numbers in the biomedical workforce; NIH peer review; and the shape of the biomedical workplace. Leadership teams spanning career stage (PhD student to Associate Dean), research approach (basic to translational), and biomedical field (social sciences to engineering) drove these pre-workshop discussions. All members of the biomedical research community were invited and two sessions were held for each topic: one at the central campus and one in the medical school. A vital feature of these pre-workshop discussions was that 30 minutes of each discussion was restricted to input from graduate students and postdocs, with the goal of ensuring frank input from trainees.

Based on these sessions, the leadership teams prepared recommendations for further discussion at the workshop itself, an all-day event in April attended by about 80 members of the UW-Madison research community, the leadership teams and organizers and several others, including Bruce Alberts (UCSF), Marc Kirschner (Harvard Medical School), Shirley Tilghman (Princeton University) and Jo Handelsman (White House Office of Science and Technology). Full details of the process, including videos and slides from the April workshop, are available at https://research.wisc.edu/biomedworkforce. The national Rescuing Biomedical Research website set up by Alberts et al. (http://rescuingbiomedicalresearch.org) also contains many useful resources.

## Workshop outcomes: core problems and symptoms of problems

The UW-Madison community was essentially unanimous about three conclusions: the biomedical research enterprise is in urgent need of an overhaul; funding for biomedical research should be increased and stabilized; and robust data and its modeling are woefully lacking but critical for making informed decisions about change. Most of our discussions focused on the overhaul, but the needs for more funding and more data were constantly brought up.

In addition to these three general conclusions, we distinguished between core problems and the symptoms of those problems, and we also identified ‘guiding themes and principles’ (Box 1) that provided a framework for our recommendations. We caution that, as with any large and diverse group of strong-minded individuals, no recommendations were unanimously supported. We also caution that biomedical scientists were not represented equally across disciplines, despite sincere efforts to have this happen. Nonetheless, what follows is based on broad input, diverse experience and a genuine desire to move the national debate forward.

10.7554/eLife.09305.003

Box 1. **Guiding themes and principles.**

Over the course of the workshop process it became apparent that the participants (despite coming from different subjects and being at different career stages) agreed on a number of themes and principles, and these guided our recommendations. These themes and principles—which are listed below—are contrary to current practices in many ways, which confirms the need for radical change in the way that biomedical research is organized and funded in the US.

1. Highly trained scientists are essential in our global economy.
2. Research and mentoring are the primary responsibilities of independent investigators.
3. PhD training equips students to be rigorous thinkers, a fundamental skill in diverse careers.
4. Scientific breakthroughs cannot be planned.
5. Sustainable research requires stable, flexible support from both federal and local institutions.
6. Research output per dollar spent should be maximized.
7. A diverse portfolio is essential for the long-term health of biomedical research.
8. Competition strengthens research, but hypercompetition weakens it.
9. Basic research provides raw material for translational advances.

**DOI:** http://dx.doi.org/10.7554/eLife.09305.003

Our process identified two core problems that the US biomedical research community faces:

- Too many researchers vying for too few dollars.
- Too many postdocs competing for too few faculty positions.

These core problems are related but distinct. The importance of this distinction becomes clear when one considers the impact of specific proposals. Curtailing graduate training might reduce competition among postdocs for faculty positions, but would not alleviate hypercompetition for grant money, at least in the foreseeable future. Conversely, increasing the NIH budget would reduce the stress for those who land faculty positions, but would not reduce the number of postdocs vying for those positions.

In contrast to these two core problems, most other issues facing the biomedical research community can be viewed as symptoms. These symptoms include: an NIH peer review system that is overwhelmed with grant applications and unable to fund deserving grant proposals; a reduced diversity of the nation's research portfolio with a resulting lowering of its potential for transformative breakthroughs; investment loss and inefficiencies due to funding instability; and investigators spending too much time submitting and re-submitting grant applications and too little time reading, thinking and mentoring students.

The distinction between core problems and symptoms is important when evaluating the impact of proposed changes to the research system. Fixing core problems will alleviate symptoms, but not the reverse. Nonetheless, alleviating symptoms will improve the research enterprise, and such changes are likely faster, easier and less controversial than core changes. Therefore both problems and symptoms should be tackled in parallel.

Our debates had one overarching theme: the biomedical research enterprise is far too valuable to our nation's health and economy to take chances on radical changes based on ideas with insufficient data. The Titanic must be turned, not capsized. Accordingly, we suggest the following recommendations to help ameliorate and eventually fix the core problems and their many symptoms. We acknowledge that many of our recommendations are not unique, but their generation from an inclusive process is unique and will, we hope, add weight to them.

## Recommendations to fix the core problem of too many researchers vying for too few research dollars

**Figure 2.**
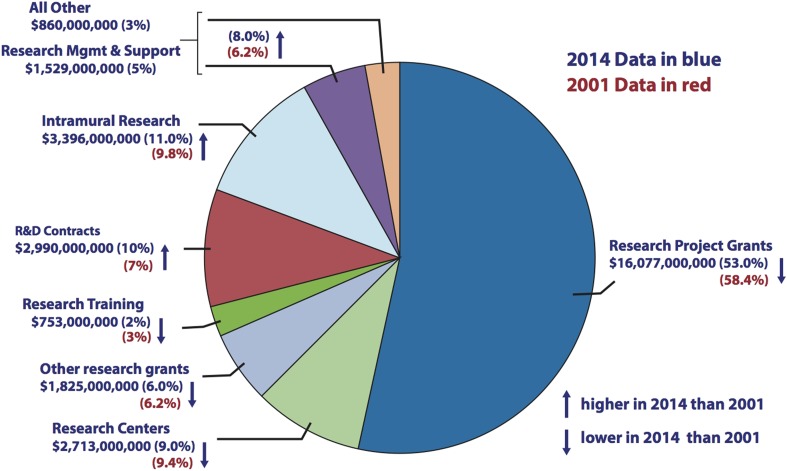
Comparison of NIH budget allocations in 2001 and 2014. Pie chart showing how the NIH spent its budget in 2014. Research Project Grants accounted for $16,077m in 2014, which was 53% of the total (blue), compared with 58.4% of the total in 2001 (red). The proportion of the NIH budget spent on R&D contracts and Intramural Research increased over the same period. Source: http://report.nih.gov/nihdatabook/index.aspx (budget history). Note: the ‘All Other’ and ‘Research Management and Support’ categories were combined in the 2001 budget. **DOI:** http://dx.doi.org/10.7554/eLife.09305.004

The success rates of investigator-initiated NIH grant applications have halved over the past 15 years—from ∼30% in 2000 to ∼15% now (FASEB, 2015), in part due to a constant increase in number of investigators and in part due to a redistribution of NIH dollars towards R&D contracts and intramural research at the NIH's own research centers (Figure 2). At the same time, NIH awards have shifted towards senior investigators at the expense of junior investigators, and towards risk-averse projects, often with a translational focus. These shifts endanger the next generation of scientists, and they also endanger research in basic science, which has historically been the engine for groundbreaking discoveries (see, e.g., MIT Committee to Evaluate the Innovation Deficit, 2015). Our recommendations are designed to reverse these trends by redistributing funds to support both junior investigators and pioneering projects. That redistribution will be painful, especially for established senior investigators, but necessary to support the next generation and cutting edge research.

First, the NIH should limit its support (from all NIH sources) for investigator salaries to a maximum of 50%, an idea endorsed by others (Alberts, 2010). This recommendation would counter the growing trend of requiring that faculty raise a significant percentage of their salary on their grants: it would also ensure that faculty expansion is accompanied by a long-term institutional commitment. Second, in addition to offering grants to fund projects, more NIH institutes should offer grants to fund investigators, such as the new MIRA award being introduced at the NIH National Institute of General Medical Sciences. When investigators select this funding route, they will spend less time writing grant proposals and more time engaging in research and mentoring trainees. Such awards will also introduce flexibility into how research funds are used, thus maximizing research output per dollar spent.

Third, NIH institutes should limit the total direct funds from all NIH sources awarded to a given lab. For example, a million dollar limit could be applied to investigators in basic biomedical research. Fourth, the NIH should increase the proportion of its budget directed to Research Project Grants, Center Grants and Training, and it should decrease the proportion directed to R&D contracts, Requests for Applications (RFAs) and intramural research. These changes would redirect funds towards investigator-initiated research and allow funding of a greater diversity of projects. R&D contracts and RFAs place limits on the topics and approaches that can be pursued, so a shift away from them will lead to fewer intellectual constraints being placed on researchers. We emphasize that this is not a recommendation to eliminate R&D contracts or RFAs, but rather to reduce their number, which will sharpen their quality and provide the funds needed to award more investigator-initiated grants. Last, the NIH should continue to set higher pay-lines for ‘early stage’ investigators and to prioritize ‘first-time renewals’ in order to promote the research of these junior investigators. Implemented together, these recommendations will free funds within the NIH budget for cutting-edge research and ensure more sustainable research programs for investigators at all career stages.

## Recommendations to fix the core problem of too many postdocs competing for too few faculty positions

The number of biomedical science postdocs in the US greatly outstrips the number of research faculty positions available. As a result, competition for independent positions has become fierce and postdoctoral training has lengthened. Previous suggestions to address this issue included a major cut in numbers of graduate students (Bourne, 2013; Alberts et al., 2014). However, this recommendation received little support from the UW-Madison research community. Cutting the number of students would limit the number of trained scientists entering the workforce, which was not favored. In addition, casting a wide net was viewed as the best way to capture the most talented and diverse graduate students, as argued previously by others (Kelly and Marians, 2014; Marder, 2014). And there was little support among faculty for the proposal to shift funding for graduate students from research project grants to training grants. There were three main reasons for this: training grants only cover a portion of training costs; they fund few non-US citizens; and the relatively narrow focus of training grants would limit intellectual diversity. The prevailing view was that dramatic cuts to graduate student numbers and a shift to training grants would do more harm than good.

Instead of cutting the number of graduate students, we recommend a narrowing of the workforce pipeline at a later stage. Our first recommendation is that fewer PhD students continue as academic postdocs. Most workshop participants preferred this option and supported the broadening of PhD programs to include experiences relevant to non-academic careers. In the trainee-only discussions, students reported feeling pressure from their mentors to pursue an academic career, and they argued that opportunities for experience with non-academic careers should be expanded to make them less likely to ‘default’ to an academic postdoc (see Box 2). This suggestion need not extend the length of graduate training, but it does require a cultural shift within some disciplines.

10.7554/eLife.09305.005

Box 2. **Input from students and postdocs.**

As UW-Madison graduate and postdoctoral trainees who attended the March pre-workshop discussions and the April workshop, we recommend the following proposals to address the systemic flaws in biomedical research in the US:

- Too many PhDs for too few PhD-worthy careers: Expand Masters programs as an honorable option within PhD training programs, which may be coupled with secondary training in law, policy, communication, business or other areas.
- Inherent conflict of interest in trainee funding: Decouple trainee support from investigator funding and provide trainees with time to pursue career-oriented training. We recommend two weeks of protected time per semester.
- Inflexible and outdated definitions of success: Broaden the metric of success used at NIH and promoted by principal investigators to recognize successful careers outside academia and reward diverse training opportunities.
- Limited knowledge of funding system among trainees: Require a course on research administration, funding mechanisms and institutional policy to educate and empower junior scientists to more effectively engage in these national discussions.

Kimberly A Haupt, Carlton P Frost, Dominique N Lisiero PhD, Patrick E Nyman, Funita P Phan, Aman Prasad, Megan E Spurgeon PhD.

**DOI:** http://dx.doi.org/10.7554/eLife.09305.005

In parallel with these changes to PhD programs, the NIH must revise its criteria for evaluating training grants so that non-academic science careers are considered as successful training outcomes for students funded by such grants. To increase transparency about the career paths open to PhDs, all NIH-funded labs and training programs should post trainee career outcomes on their websites. Grant holders should also be required to be supportive of trainees who want to pursue careers outside research. This pipeline change will not reverse the current crisis immediately but it should have a major impact within a few years. A second mechanism is for a subset of graduate students to earn a Masters of Science degree instead of a PhD. One problem with this suggestion is that a Masters degree is sometimes viewed as a consolation prize, rather than a valuable postgraduate qualification: to combat this perception, we suggest that a Masters degree should be made a mandatory step towards a PhD.

Our second recommendation is to increase the number of scientists who have a supporting role, rather than the leading role, in a lab: the idea of increasing the number of such ‘staff scientists’ has also been proposed by others (Alberts et al., 2014; Daniels, 2015). Such a shift has the dual advantage of reducing reliance on trainee labor and improving career prospects in biomedical research. But that shift comes with increased expense and a need for more stability of the staff scientist career track, a concern voiced by UW-Madison staff scientists (see Box 3). We suggest a few mechanisms to foster this career track. First, the NIH should provide funds to cover the extra cost of replacing a trainee position with a staff scientist; the mechanism for doing this could be similar to the supplements used to promote the diversity of the research workforce. Second, the NIH should expand the number of Research Specialist Awards recently introduced at the National Cancer Institute (NCI) to support staff scientists. Finally, institutions should develop mechanisms to recognize staff scientists and promote their careers.

10.7554/eLife.09305.006

Box 3. **Input from staff scientists.**

We believe that an increasing reliance on staff scientists is an attractive way to redress the imbalance between the number of PhDs and the opportunities for PhDs in research. However, academic staff scientist positions often lack stability and opportunity for advancement. Accordingly, we recommend that the stability of the staff scientist position should be increased progressively with time of employment by the development of federal or institutional funding mechanisms that cover at least part of the salary of the staff scientist. We also recommend that institutional rules be modified to explicitly increase the opportunities open to staff scientists for promotion, recognition and compensation. Additionally, we recommend that staff scientists be allowed multiple roles as a means to both broaden the work experience of the scientists and to provide a potential fallback if support for research is lost: these other roles could include administration (e.g., at a core facility), teaching or outreach. Finally, we recommend that the roles and expectations of staff scientists be clearly defined.

Andrea Bilger PhD, Mats W Johansson PhD, Suzanne M Ponik PhD, Megan E Spurgeon PhD.

**DOI:** http://dx.doi.org/10.7554/eLife.09305.006

Our final recommendation is that institutions should increase incentives for retirement. We did not favor an NIH emeritus award to support lab closure, because such awards take funds from more competitive research, and we did not favor NIH grants explicitly partnering senior and junior investigators, because forced collaborations rarely yield innovative science. Instead, institutions should remove barriers to retirement and provide incentives for lab closure as a way to open more faculty positions.

## NIH peer review: reducing pressure on the system

Pressures on NIH peer review have never been greater. The number of NIH grant applications has doubled over the past 15 years, while funding has remained flat and research costs have skyrocketed. No one doubts that the historically low success rate of NIH awards has created tremendous strain on the peer review system and on the biomedical research enterprise more broadly. We suggest a three-pronged approach to address these pressures and improve the current system.

First, the NIH should take steps to reduce the number of grant applications. These steps should include: capping the number of grant submissions per investigator per year (with exceptions for unfunded investigators); reducing the turnaround time between submission and funding to six months, so that fewer applications are submitted as ‘back-ups’; and, as mentioned above, introducing more investigator-based rather than project-based grants. More radically, we suggest that the NIH raise the pay-line to 20%, regardless of the funds available, with a sliding scale of funding that depends on the score received by the application and on the other funds available to the investigator. For example, those with higher scores might receive close to their full request, while those with lower scores might receive only half the amount requested (and be expected to achieve half of the aims listed in the application). An increased pay-line will reduce the number of applications because strong proposals will not require re-review. It will also decrease the lottery-like, all-or-nothing aspect of grant review, increase funding stability at the expense of lab size, and broaden the NIH's research portfolio. Collectively, the recommended changes will result in investigators spending less time applying for funding and more time conducting research and mentoring. They would also allow staff at the NIH Center for Scientific Review to spend less time organizing reviews and more time training reviewers.

Second, the NIH must take steps to ensure a high standard of peer review, a goal that becomes more tractable with higher pay-lines and fewer grant proposals. We recommend that all NIH-funded investigators be required to serve as reviewers if asked; that performance reviews be established for study-section chairs, grant reviewers and NIH Scientific Review Officers; and that reviewer terms be shortened to three years. In addition, we suggest that the NIH reduce the number of grant cycles, ideally to two per year, which should be possible with changes that result in fewer applications and a shorter turnaround time (as described above). We also emphasize that study sections should maintain diversity with respect to geographical region, gender, ethnicity and academic rank.

Our third major recommendation is that peer review be revamped to emphasize the importance of untargeted discovery-driven science. We first suggest that a new track be established for innovative proposals. These proposals should be short with online reviews by scientists who themselves have a track record of innovation, and the turnaround time between submission and funding should be short (ideally just three months). We envision a monthly review process in which 10 reviewers, covering broad research areas, each respond yea or nay to a proposal, with one or two explanatory sentences, and with a final vote of >70% approval required for funding.

We also encourage the NIH to bolster recent efforts to eliminate the use of translational relevance for judging basic science proposals. Instead, such proposals should be judged on how well they advance a fundamental understanding of biological mechanisms. Finally, we urge that the NIH discontinue the practice of distributing funds as a percentage of the number of grants submitted in a particular area. This policy can overfund areas in which there are many proposals taking similar approaches, and where in some cases the projected outcomes are of relatively low significance, and underfund other areas in which there are fewer proposals but the projected outcomes could be more groundbreaking. Instead, the NIH should evaluate research across study sections and award funding on the basis of significance, quality and originality of the expected outcome.

## The shape of the biomedical research workplace: doing more with less

The imbalance between researchers and research dollars is placing tremendous stress on the research workplace. We suggest three changes to the workplace that will foster state-of-the-art research with fewer resources and increase the research output per dollar spent. First, institutions must improve core facilities. Mechanisms should be established for the following: to evaluate cores regularly and coordinate core services; to set priorities for investing in new and existing cores; and to decommission cores no longer needed. Centralized, online information about core facilities on each campus will make them more accessible. To provide stability for these key resources, funding of core facilities and their staff scientists should be a joint venture between institutions and the NIH via infrastructure grants, indirect costs and other sources.

Second, institutions must change their workplace structure to accommodate a diversity of lab formats. Individual labs funded by single investigators have been the norm and remain tremendously successful, but collaboration is increasingly necessary, in part because of the development of highly specialized methods and equipment that cannot be housed within a single lab, and in part as an attractive way to create a critical mass of researchers despite shrinking funds. Therefore, institutions should consider ways to create flexible research space to help foster interactions.

Third, barriers to collaboration must be removed, particularly for assistant professors. Despite many research fields becoming heavily collaborative, assistant professors sometimes avoid collaborations to prove their independence in order to achieve tenure. In these fields, avoiding collaboration hamstrings junior investigators. We specifically suggest that tenure-granting institutions develop explicit guidelines for evaluating collaboration (both in publications and in funding). Another barrier to collaboration is that junior investigators who join a multi-investigator grant can lose their early-stage investigator status (and the higher pay-line associated with it). The NIH should correct this policy.

## Funding for biomedical research should be increased and stabilized

We need to start fixing the systemic flaws in the biomedical research enterprise now, whether or not the NIH receives additional funds. Yet additional funds are also critical to stabilize and sustain our decades of investment in biomedical research. US spending on research as a percentage of its GDP has dropped precipitously over the past two decades, from second to tenth among developed nations (American Academy of Arts and Sciences, 2014).

Scientists of all career stages must become engaged in efforts to communicate with the public and with Congress as continued public and political support are needed to ensure that biomedical research is funded at the level required to deliver groundbreaking medical advances over the next decade and beyond. Competition for discoveries in biomedical research is global, and winners will be rewarded in the long term with healthier societies and stronger economies. Cultural changes can and must strengthen biomedical research, but a sustained increase in federal funding is also essential as research costs continue to increase and opportunities expand. However, we caution against a massive short-term increase and instead recommend that the US government introduce legislation to ensure that a fixed percentage of GDP is spent on biomedical research: this would eliminate the boom-and-bust cycles of research funding that can be both damaging and wasteful (FASEB, 2015).

## Moving forward: what's next?

The UW-Madison workshop was part of a larger national movement to address systemic flaws inherent to the current biomedical research enterprise. We encourage others to organize workshops that bring together diverse elements of the research community and give voices to junior colleagues whose future is our future. We anticipate that additional workshops with campus-wide participation will advance the national debate and help individual campuses to put the plethora of recommendations in context and to generate new ones. An unavoidable conclusion of our workshop is that no single solution will be effective, but that a constellation of changes is needed to reverse current trends and strengthen biomedical research. The problems we face are large and complex, so having greater numbers of creative and invested minds focused on them will lead to more and better ideas for solutions.
